# Confronting the Emerging Threat to Public Health in Northern Australia of Neglected Indigenous Arboviruses

**DOI:** 10.3390/tropicalmed2040055

**Published:** 2017-10-17

**Authors:** Narayan Gyawali, Andrew W. Taylor-Robinson

**Affiliations:** 1 School of Health, Medical & Applied Sciences, Central Queensland University, Rockhampton, QLD 4702, Australia; n.gyawali@cqu.edu.au; 2 Institute of Health & Biomedical Innovation, Queensland University of Technology, Brisbane, QLD 4059, Australia; 3 School of Health, Medical & Applied Sciences, Central Queensland University, Brisbane, QLD 4000, Australia

**Keywords:** arbovirus, neglected, undifferentiated febrile illness, Northern Australia, diagnostics, control, prevention

## Abstract

In excess of 75 arboviruses have been identified in Australia, some of which are now well established as causative agents of debilitating diseases. These include Ross River virus, Barmah Forest virus, and Murray Valley encephalitis virus, each of which may be detected by both antibody-based recognition and molecular typing. However, for most of the remaining arboviruses that may be associated with pathology in humans, routine tests are not available to diagnose infection. A number of these so-called ‘neglected’ or ‘orphan’ arboviruses that are indigenous to Australia might have been infecting humans at a regular rate for decades. Some of them may be associated with undifferentiated febrile illness—fever, the cause of which is not obvious—for which around half of all cases each year remain undiagnosed. This is of particular relevance to Northern Australia, given the Commonwealth Government’s transformative vision for the midterm future of massive infrastructure investment in this region. An expansion of the industrial and business development of this previously underpopulated region is predicted. This is set to bring into intimate proximity infection-naïve human hosts, native reservoir animals, and vector mosquitoes, thereby creating a perfect storm for increased prevalence of infection with neglected Australian arboviruses. Moreover, the escalating rate and effects of climate change that are increasingly observed in the tropical north of the country are likely to lead to elevated numbers of arbovirus-transmitting mosquitoes. As a commensurate response, continuing assiduous attention to vector monitoring and control is required. In this overall context, improved epidemiological surveillance and diagnostic screening, including establishing novel, rapid pan-viral tests to facilitate early diagnosis and appropriate treatment of febrile primary care patients, should be considered a public health priority. Investment in a rigorous identification program would reduce the possibility of significant outbreaks of these indigenous arboviruses at a time when population growth accelerates in Northern Australia.

## 1. Introduction

*Ar*thropod-*bo*rne*viruses* (arboviruses) are by definition transmitted between vertebrate hosts by biting arthropods (mosquitoes, ticks, sandflies, midges and gnats) [1], and the infections that they cause pose a significant public health risk worldwide. The International Catalogue of Arboviruses currently lists 537 registered viruses on the basis of their known transmission by arthropods, known for potential infectivity to humans or domestic animals, and antigenic or phylogenetic relationships to known arboviruses [2].

At present, more than 130 arboviruses are recognised as causing mild to fulminant disease in humans [3]. Symptoms of uncomplicated arboviral infection generally occur between 3 and 15 days after exposure to the virus and may persist for a week or so. The most common clinical features of infection are the indistinct influenza-like symptoms of fever, headache, and malaise, which, without recourse to further information regarding a patient’s clinical and exposure history, often preclude a correct diagnosis [4].

Australia is home to over 75 arboviruses that have been isolated from its native arthropods [2]. While so far only 13 of these are found to be associated with human infection, just Barmah Forest virus (BFV) and Ross River virus (RRV) are tested for routinely. Moreover, laboratory tests are available for Murray Valley encephalitis virus (MVEV) and West Nile Kunjin virus (KUNV) but test requests are made on patients with highly suggestive signs and symptoms [5]. The ecology and role of other arboviruses in humans, whether they are associated with any serious infections or undiagnosed undifferentiated febrile illness (UFI), are unknown and their study is not prioritised. An analysis of the notifications of BFV, RRV, MVEV, and KUNV in the last two decades has clearly shown a higher distribution of these viruses in Northern Australia [6] (reviewed in [5]).

In this article, we describe briefly the neglected Australian arboviruses that are most likely to emerge as significant agents of human disease. The arboviruses that we discuss have been found to infect humans—serological evidence of host immune responses has been found. It is implicitly understood that a virus that is associated with human infection could potentially be a pathogen, i.e. it may have been causing a disease, the aetiology of which is so far unknown, or it could cause disease under certain circumstances, such as in immunocompromised persons, during pregnancy, or upon secondary infection. We also consider what action should be taken to confront the potential threat of such neglected indigenous arboviruses in the particular environment of Northern Australia. This is a largely tropical climatic region where both mosquito vectors and vertebrate reservoir hosts are abundant and in which a future major expansion of a human population primarily comprising relocating, previously non-exposed individuals, is predicted.

## 2. Arbovirus Ecology and Epidemiology

Most arboviruses studied thus far are transmitted in zoonotic cycles, i.e. the principal vertebrate host is an animal other than human [7]. The distribution of an arbovirus is restricted to areas inhabited by vertebrate hosts that serve as its reservoirs and vectors. Thus, many arboviruses have clearly defined ecological zones, while some, distributed globally, cause diseases of considerable public health and veterinary importance (reviewed in [8]). Examples of the latter include dengue (worldwide, approximately between the Tropics of Cancer and Capricorn), yellow fever (Africa and South America), Japanese encephalitis (eastern and southeast Asia and Australia), West Nile encephalitis (North America, Europe and the Middle East), chikungunya (Asia, Central and South America, parts of the Pacific), eastern and western equine encephalitis (North America), and Venezuelan equine encephalitis (South America). Due to focal, global, environmental, societal and/or demographic changes, many of these viruses have either emerged or re-emerged in the first years of this century [9,10,11].

Notably, the non-segmented, positive-strand RNA viruses belonging to the genus *Flavivirus*, family *Flaviviridae*, or genus *Alphavirus*, family *Togaviridae*, are the aetiological agents of several major global infectious diseases such as dengue, yellow fever, chikungunya and Zika. Other related pathogens belong to the segmented, negative strand RNA *Orthobunyavirus* genus. The vast majority of arbovirus-associated epidemics occur in the tropics and subtropics due to the prevailing hot and humid climate which is conducive to the habitation of vector mosquitoes, including members of *Aedes, Anopheles, Culex*, *Haemagogus,* and *Ochlerotatus* genera [12]. To this growing list of real or potential public health threats posed by arboviruses Mayarocan now be added, identified recently in the Amazon and other tropical regions of South America [13]. The issue of whether neglected Australian arboviruses similarly present an emerging, hitherto unrecognised challenge to humans is a subject of discussion.

## 3. Arboviruses in Australia

Australia is the sixth largest country in the world by area, the largest country without land borders, and the largest country overall in the southern hemisphere. Early European settlement, urbanisation, increased sea and air travel and trade, globalisation, pathogen evolution, and elevated mean global temperature are some of the factors that may have influenced the introduction and expanded geographical reach of infectious diseases, including those caused by arboviruses, in Australia [14]. Furthermore, Australia spans tropical and subtropical latitudes, where arboviruses have access to an abundant source of both reservoir hosts and vectors. 

**Figure 1:**
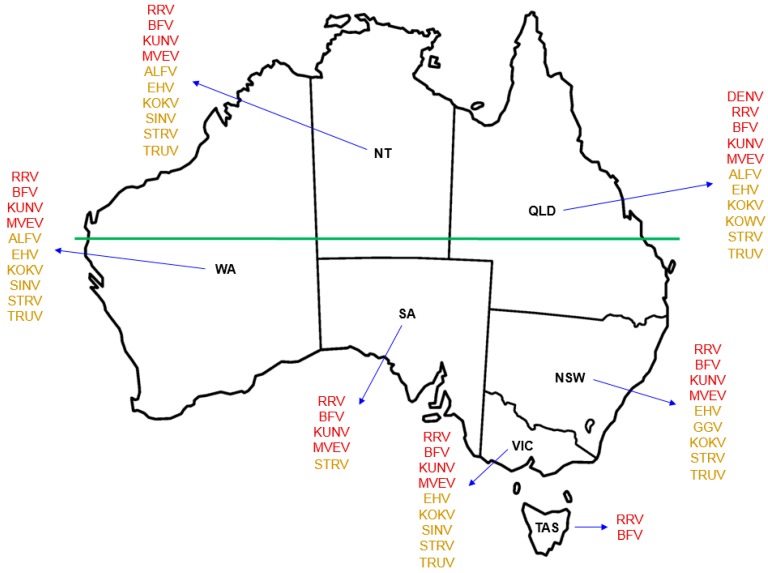
Geographical distribution of Australian indigenous arboviruses known to cause human infection. Use of red font for each named virus indicates the state or territory from which that virus is known to be recovered and the notifiable disease for which it is listed in the Australian National Notifiable Disease Surveillance System (ANNDSS). Use of amber font for each named virus indicates the reported recovery of that virus from mosquitoes during mosquito surveillance but that the corresponding virus-associated disease is not currently recorded in the ANNDSS. Named arboviruses: ALFV—Alfuy; BFV—Barmah Forest; DENV—Dengue; EHV—Edge Hill; GGV—Gan Gan; KOKV—Kokobera; KOWV—Kowanyama; KUNV—Kunjin; MVEV—Murray Valley encephalitis; RRV—Ross River; SINV—Sindbis; STRV—Stratford; Trubanaman—TRUV. The land mass above the horizontal green line, which marks the southern edge of the Pilbara Range (latitude 24° S, just south of the Tropic of Capricorn, 23.52° S), approximates to the region termed Northern Australia. States and territory: NSW—New South Wales; NT—Northern Territory; QLD—Queensland; SA—South Australia; TAS—Tasmania; VIC—Victoria; WA—Western Australia.

While only 13 of the more than 75 identified arboviruses indigenous to Australia are currently known to cause disease in humans, information is scarce as to the potential human pathogenicity of most others [15]. Of those that are recognised to cause infection in humans in Australia (Figure 1), the alphaviruses RRV and BFV are the most well-known, infection with either of which triggers an incapacitating and occasionally chronic polyarthritis with accompanying myalgia and lethargy [16,17]. The flaviviruses MVEV and KUNV cause encephalitis, an acute inflammation of the brain [18].

Infection with the flavivirus dengue (DENV) is typically characterised by a febrile illness but a small proportion of cases manifest as a life-threatening haemorrhagic fever or shock syndrome (reviewed in [19]). DENV may be acquired outside Australia and brought back by returning travellers, a significant proportion of whom are hospitalised with unrecognised warning signs of severe disease. As intercontinental travel from Australia, particularly to Asia, continues to increase, in order to avert serious outcomes it is crucial that clinicians anticipate, and can recognise and manage, such tropical infectious diseases. While DENV has a transglobal distribution, local outbreaks are also reported regularly in far north Queensland, with foci in the vicinities of Cairns and Townsville [20], where it is well recognised by the resident population as a not insignificant threat to their health [21].

Several other arboviruses that are indigenous to Australia (Figure 1), such as the alphavirus Sindbis (SINV), the flaviviruses Alfuy (ALFV), Edge Hill (EHV), Kokobera (KOKV) and Stratford (STRV), and the orthobunyaviruses Gan Gan (GGV), Kowanyama (KOWV) and Trubanaman (TRUV), are recognised through eliciting mild symptoms of febrile illness, corroborated by detection of serum antibodies to viral antigens, as being able to infect humans [17,22,23] (reviewed in [5]). There are occasional reports of human disease caused by SINV, EHV and KOKV [24,25,26], but these are not currently included individually in the list of Australian national notifiable diseases by disease type [27]. The magnitude of each of these arboviral infections raises the question as to what is an appropriate threshold for recording cases for the purposes of annual notification at state/territory and national levels. SINV is reportedly the arbovirus most frequently isolated from mosquitoes in Australia [23], but as an alphavirus it does not come under the ‘flavivirus infection (unspecified)’ umbrella presently used for nationwide notification [27].

Other arboviruses have been isolated from arthropods in the Australia-Pacific region [15]. These include the newly identified Bamaga (BGV) and Fitzroy River (FRV) flaviviruses [28,29], which are closely related to the disease-causing yellow fever virus (YFV) and EHV, but for each of which there is scant information about its capacity to infect humans or to cause disease in humans.

## 4. Undifferentiated Febrile Illness and Pyrexia of Unknown Origin

Fever, defined as an abnormally high body temperature (>100 °F, 37.8 °C), is a common symptom of patients seeking healthcare. Due to the non-specific clinical manifestations and a lack of positivity in initial laboratory testing, the cause of fever may not be identified. When the onset of fever is acute and no cause can be found after taking a full history and physical examination of the patient, it is called a UFI. If the UFI continues, it is classified as a pyrexia of unknown origin (PUO), defined in 1961 as an illness of more than three weeks’ duration, with fever greater than 101 °F (38.3 °C) on several occasions, the cause of which is not identified after one week of in-hospital investigation [30]. Since this description does not include many self-limiting viral diseases, it was revised in 1991 [31]. The newer definition of PUO has four categories: classical; hospital-acquired; neutropenic (immune-deficient); and HIV-associated. Also, the revision proposed a minimum of three days of hospitalisation or at least three outpatient visits before this diagnosis may be made. Most commonly, PUO is the result of infection, malignancy, or non-malignant inflammatory diseases [32]. 

## 5. UFI/PUO as a Health Problem

Between 20% and 60% of UFI cases are attributed to infections [31,33,34,35]. The aetiological agents of UFI and PUO vary according to the geography and demography of the patients. For instance, in post-industrial countries, self-limited viral infections and infections with bacteria such as *Brucella* spp., *Leptospira* spp., and the atypical mycobacteria are major causes of UFI/PUO. In economically emerging nations, UFI/PUO include illnesses caused by a diverse range of human pathogens including *Mycobacterium tuberculosis*, *Neisseria meningitidis*, systemic *Salmonella enterica* infections, *Plasmodium* spp., DENV, Epstein-Barr virus, cytomegalovirus, and hantaviruses [36,37,38]. 

In a landmark prospective study in Belgium of patients hospitalised with febrile illness, depending on if and when a final diagnosis was in fact established, an estimated 12–35% were assessed to have died from PUO-associated complications [39]. The cause of the fever remained obscure in 48% of patients with episodic fever, compared to 26% of patients with continuous fever [39]. Prolonged febrile illnesses remain a diagnostic challenge; about one-third to half of PUO cases remain undiagnosed [40,41,42]. In developing countries, a diagnosis of UFI/PUO may result from a lack of laboratory resources but even in a high-income nation like Japan that has excellent diagnostic tools, 28.9% of PUO goes undiagnosed [43]. 

## 6. Diagnosis of Australian Arboviral Infection

For almost a decade after the identification of RRV in 1959 [44], only small numbers of patients were identified as having a clinical infection with this agent, because virological and serological diagnostic testing was available only within a research framework using an in-house test. Following the development of a commercial enzyme-linked immunosorbent assay (ELISA) to detect anti-RRV immunoglobulin (Ig)M antibody [45], the number of patients diagnosed annually rose to between 4000 and 6000 [46]. The number of localities from where RRV cases were reported increased almost two-fold from 1985 onwards [47].

Following its identification from northern Victoria in 1974 [48], a similar experience occurred with the diagnosis of BFV infection and its annual notification [49]. Epidemic polyarthritis, the now outmoded term that was then used to describe the autoimmune conditions associated with both RRV and BFV, became a nationally notifiable disease in 1990 [46]. While typically there are around 4500 notifications of epidemic polyarthritis per annum, 9554 cases were reported in 2015 [50].

Clinical infections with KUNV [51,52,53,54], EHV [25], GGV and KOKV [22,26] can now be confirmed in specialised laboratories, but only suspected KUNV infected cases undergo screening as standard.

## 7. A Causal Link between Neglected Arboviral Infections and UFI/PUO?

It has been proposed that arboviruses may be responsible for some cases of UFI observed in Australia [55]. While remarkably few systematic studies of UFI or PUO in an Australian setting have been undertaken, those that have been performed suggest that a large proportion of UFI/PUO cases remain undiagnosed (reviewed in [5]). This is despite the now-routine commercial testing for RRV and for BFV. A three-year retrospective study from 2008–2011 of a tertiary referral hospital in North Queensland found 58.8% of patients with UFI had no definitive diagnosis [56]. Neglected indigenous arboviruses may have infected humans regularly for decades, thereby being responsible for at least some of these UFI cases in this tropical north region. The possibility of arbovirus pathogens from Northern Australia causing more wide-scale outbreaks, such as the notified incidences of MVEV in 2001, 2008 and 2011, and the KUNV equine outbreak of 2011 in south-eastern Australia [57], should also be considered. While the horse-derived WNVNSW2011 strain of KUNV not only differed to, but was more virulent than, other KUNV strains that circulated previously in Australia [57], it may be argued that the ecology of this arbovirus changed alongside the emergence of virulence.

The introduction of commercial screening for RRV and BFV led to a highly significant rise in their respective reported rates of infection when compared to historical records [46,49]; these conspicuous examples of unforeseen prevalence may also apply to other arboviral infections. Hence, it is possible that further, neglected, arboviruses—for which diagnostic tests are not yet available outside research laboratories—are a major underlying cause of undiagnosed UFI/PUO cases in Australia.

## 8. Transmission Cycles of Australian Arboviruses

Over several decades, many arboviruses have been identified in Australian mosquitoes, ticks, and biting midges [5,15]. Little is known about their transmission cycles, their pathogenicity for humans, or their potential to cause epidemics. Although large marsupials such as kangaroos and wallabies are considered potential reservoirs for RRV [58,59] and BFV [59,60], and waterbirds such as herons and egrets are regarded as hosts for MVEV, ALFV and SINV [61,62], there are many other arboviruses whose relationship with reservoirs and vectors, and their role in human infections or diseases, are yet to be defined.

While the epidemiology of these arboviruses is poorly understood, it is likely that they are maintained in zoonotic cycles rather than by human-to-human transmission. It may be that these neglected viruses are harboured by apathogenic, persistent infections in native Australian reservoir mammals and birds, with occasional spillover into humans [5]. 

## 9. Northern Australia’s Climate Favours Arboviruses

Many of Australia’s indigenous arboviruses that are known to cause human disease have been recovered from Northern Australia (Figure 1). Since it had no previous political purpose, the term ‘Northern Australia’ was defined formally only very recently with the passing of the Northern Australia Infrastructure Facility Act 2016 [63]. Although there are several minor qualifications, broadly speaking it is considered to comprise the Northern Territory and the areas of Queensland and Western Australia that are north of the Tropic of Capricorn (latitude 23.5 degrees south of the Equator). 

The northern coastal fringe of the country is made up of northern Queensland, the Northern Territory, and the remote Kimberley and Pilbara Ranges of Western Australia. Uniquely for Australia, the region experiences a tropical, often monsoonal, wet season during the southern hemisphere summer months of November to April each year [59,64]. Moreover, if the mean annual air temperature continues to rise as a consequence of global climate change, the spatial range of mosquito species able to transmit arboviruses is likely to broaden [65]. While the presence of vectors does not necessarily mean the emergence of human pathogens, these factors contribute to favourable breeding conditions for mosquito species that are especially well-suited to maintaining arboviruses of potential public health importance [66].

## 10. Potential Public Health Threat

The Australian Commonwealth Government is actively promoting increased settlement and economic activity in the currently less populated areas that lie to the north of the Tropic of Capricorn as an integral part of its ‘Developing Northern Australia’ white paper for massive infrastructure investment in this region over the coming decades [67]. Although it comprises nearly half of the total land mass of the country, Northern Australia includes only about one-quarter of the current Australian population. It is therefore considered to be a region of largely untapped potential that is ripe for 21st century population growth outside of the urban densification in the major metropolitan conurbations to the south [67]. An incentivised expansion of the industrial, business and agricultural development of this vast tract of land is predicted, with an increase in the residential population from the current 1.33 million to up to 2.9 million people by 2050 projected [68]. The anticipated increased human activity in many areas of the tropical north of Australia will lead to fast-growing urbanisation that places relocated immune-naïve people into closer proximity to native reservoir wildlife, as well as to vector mosquitoes, for Australian indigenous arboviruses.

The growth in agriculture and other economic developments proposed for these localities will inevitably alter the ecology of the native animals and birds that act as reservoir hosts for numerous neglected Australian arboviruses, as well as affecting the mosquito vectors [5]. Additionally, sudden climatic and environmental variations [69], including the high rainfall, more frequent cyclones and resultant increased intensity of flooding associated with outbreaks of MVEV [70] and RRV [71], have occurred with alarming regularity in recent years [72], potentially generating an ecological expansion of Australian arboviruses. These circumstances therefore create a perfect storm for greater prevalence of infection with neglected Australian arboviruses, particularly in the tropical north of the country. It is perhaps worth considering that notable close relatives of these many indigenous arboviruses have already caused global pandemics in recent decades [73]. 

## 11. A Call to Arms for Novel Diagnostic Tests and Therapy Targets

In this circumstance, therefore, there is a pressing obligation to determine the geographical range and true disease burden of neglected indigenous arboviruses in Northern Australia. This may be accomplished by implementing a scheme of systematic, continual surveillance of vectors, reservoirs and viruses in order to address where, when, and how virus transmission to humans occurs as well as building up a picture of its likely impact. This may also be progressed through performing routine testing by designated public health laboratories of a systematic sub-sample of UFI/PUO patients for evidence of recent infection with neglected arboviruses as well as other potential causative agents of UFI/PUO. Furthermore, to screen patients with UFI/PUO and other suspected cases of arboviral infection, in addition to serology testing, the development of novel diagnostic tools should be given high research priority. Already available methods of detection of pan-alphaviruses and pan-flaviviruses include IgM antibody-based ELISA, quantitative reverse transcription PCR (RT-qPCR), and microarray [74,75,76]. Other state-of-the-art methods, for example RNA-seq metagenomics, which reveal an individual’s virome [77], could also be applied to this setting.

Notwithstanding the striking exceptions of YFV, Japanese encephalitis virus, and tick-borne encephalitis virus [78], an obstacle to the successful control of infections caused by arboviruses is the lack of effective, authority-registered vaccines [79]. Strenuous efforts to yield a commercially available vaccine against DENV are ongoing but these are exacerbated by media-fuelled concerns over suitability and side-effects in pilot immunisation programs [80,81]. Also, the phenomenon of antibody-dependent enhancement of infection of humans that has been shown for many flaviviruses and alphaviruses [82] is an impediment to any future potential consideration of therapeutic antibodies as an alternative treatment [83]. Given this scenario, there is a dire need to accelerate the quest for novel options for both diagnosis and therapy.

Therapy regimens that are syndrome-based are currently common practice, frequently informing the prescription of antibiotics in empirical treatment. Such antibacterial pharmaceutical agents are ineffective when the UFI/PUO is caused by arboviruses; indeed, their inappropriate use may contribute to the worsening problem of antimicrobial resistance. Early, on-site, and rapid screening for neglected Australian arboviruses could help to identify the cause of infection and thus reduce the often ill-informed perceived obligation to provide antibiotics. Adoption of this measure would also expedite early detection of outbreak foci, thereby facilitating a prompt, efficient and proportionate response. This would have the effect of limiting the spread of disease, as hindsight suggests public health policymakers could have achieved better during the recent epidemic in Latin America of the flavivirus Zika [84,85].

The existing funding model for diagnostic pathology services in Australia does not foster requests by a general practitioner or hospital clinician to test for infection with a little-known arbovirus, even if they are aware of its possible role in disease. Hence, many UFI/PUO cases are not diagnosed correctly as the treating clinicians may consider the cost of testing is not warranted or because samples for testing were collected at an inappropriate time or from an incorrect site. They also may go undiagnosed on account of the causative agent being novel, not known to cause human disease, or because there are no routine diagnostic tests available. 

For cases of UFI/PUO, for reasons of both practical feasibility and cost, it is not a realistic proposition to recommend multiple, individual laboratory tests in order to detect most or all neglected arboviruses. In light of this, development of a generic assay that would provide for many pathogens and which may be applied in a broad range of settings should be prioritised. For example, routine testing by designated public health laboratories of a two-step protocol could be envisaged, starting with pan-flavivirus and pan-alphavirus IgM antibody rapid tests and, as required of a sub-sample of patients, followed by confirmatory detection of viral RNA by RT-qPCR [76]. Along with the ability to screen for multiple arboviruses in a short space of time there is a saving in resources for the testing laboratory by virtue of a quicker diagnosis. This means that any future decision not to request sample analysis may ultimately prove a false economy.

## 12. One Component of a ‘One Health’ Approach to Combating Arboviruses

The One Health approach is a currently promulgated systems-based movement in which biomedical researchers and professionals in public health, veterinary medicine, and ecology combine their expertise in order to monitor and control the threat of infectious diseases and determine how pathogens spread among people, animals, and the environment [86]. Involvement of biomedical researchers, pathologists, and clinicians in this transdisciplinary model may lead to more efficient diagnosis of, and improved outcomes for, patients with arboviral infections.

In order to achieve success in preventing outbreaks of neglected arboviruses within the context of Northern Australia, it will be necessary to engage all relevant stakeholders, from federal, state and local authorities, via tertiary care and general practice centres, to local neighbourhoods, schools, and households. Risk of outbreak is always amplified when people are unaware of a disease or its route of transmission. As with the ongoing threat posed by DENV in Queensland [21], raising awareness levels among residents of regional communities is an extremely important component of a future public health policy for Northern Australia. Well-targeted information campaigns would aim to increase individual knowledge of the symptoms and possible sequelae of UFI/PUO and, with regard to mosquito transmission of arboviral infections, personal preventive methods and vector control.

## 13. Conclusions and Future Directions

For the neglected arboviruses that are indigenous to Australia there is an inadequate understanding of their distribution, epidemiology, and transmission ecology. Information is also lacking with respect to theimmunopathology and true disease burden, including undiagnosed cases UFI/PUO, which they cause. This knowledge gap exists despite the potential for these neglected arboviruses to become significant human pathogens in the rapidly developing region of Northern Australia, thereby presenting a major challenge to the public health of the nation, and conceivably also globally [87]. Future research into the areas discussed herein, combined with production of diagnostic tools to include first-line screening of a suite of indigenous arboviruses, would help greatly to limit the impact of this emerging threat to human health and wellbeing in the tropical north of Australia. Preferably, this would form a key component of a holistic, transdisciplinary strategy to improve environmental health in order to prevent mosquito-borne diseases in Northern Australia [88].
